# Quality and quantity of serious violent suicide attempts during the COVID-19 pandemic

**DOI:** 10.3389/fpsyt.2022.927696

**Published:** 2022-07-19

**Authors:** Tazio Maleitzke, Dario Zocholl, Tobias Topp, Annika Dimitrov-Discher, Elly Daus, Gabriel Reaux, Malin Zocholl, Rolf Nicolas Conze, Moritz Kolster, Philipp Weber, Florian Nima Fleckenstein, Louise Scheutz Henriksen, Ulrich Stöckle, Thomas Fuchs, Denis Gümbel, Nikolai Spranger, Alexander Ringk, Sven Märdian

**Affiliations:** ^1^Charité – Universitätsmedizin Berlin, corporate member of Freie Universität Berlin and Humboldt-Universität zu Berlin, Center for Musculoskeletal Surgery, Berlin, Germany; ^2^Berlin Institute of Health at Charité – Universitätsmedizin Berlin, Julius Wolff Institute, Berlin, Germany; ^3^Berlin Institute of Health at Charité – Universitätsmedizin Berlin, BIH Biomedical Innovation Academy, BIH Charité Clinician Scientist Program, Berlin, Germany; ^4^Charité – Universitätsmedizin Berlin, corporate member of Freie Universität Berlin and Humboldt-Universität zu Berlin, Institute of Biometry and Clinical Epidemiology, Berlin, Germany; ^5^Charité – Universitätsmedizin Berlin, corporate member of Freie Universität Berlin and Humboldt-Universität zu Berlin, Department of Orthopaedic, Trauma, Hand and Reconstructive Surgery, Berlin, Germany; ^6^Department of Orthopaedic, Trauma, Hand and Reconstructive Surgery, Vivantes Hospital Friedrichshain, Berlin, Germany; ^7^Department of Trauma and Orthopaedic Surgery, BG Klinikum Unfallkrankenhaus Berlin gGmbH, Berlin, Germany; ^8^Center for Orthopaedics and Trauma Surgery, Helios Klinikum Berlin-Buch, Berlin, Germany; ^9^Department of Growth and Reproduction, Copenhagen University Hospital – Rigshospitalet, Copenhagen, Denmark; ^10^International Centre for Research and Training in Endocrine Disruption of Male Reproduction and Child Health (EDMaRC), Copenhagen University Hospital – Rigshospitalet, Copenhagen, Denmark; ^11^Department of Growth and Reproduction, Rigshospitalet, University of Copenhagen, Copenhagen, Denmark

**Keywords:** injury, death, mortality, SARS-CoV-2, violent suicide attempts, COVID-19

## Abstract

**Background:**

While repeated shutdown and lockdown measures helped contain the spread of SARS-CoV-2 during the COVID-19 pandemic, social distancing and self-isolation negatively impacted global mental health in 2020 and 2021. Although suicide rates did reportedly not increase during the first months of the pandemic, long-term data, and data on the quality of serious violent suicide attempts (SVSAs) are not available to date.

**Materials and methods:**

Orthopaedic trauma patient visits to the emergency department (ED), ED trauma team activations, and SVSAs were retrospectively evaluated from January 2019 until May 2021 in four Level-I Trauma Centers in Berlin, Germany. SVSAs were assessed for suicide method, injury pattern and severity, type of treatment, and length of hospital stay.

**Results:**

Significantly fewer orthopaedic trauma patients presented to EDs during the pandemic (*n* = 70,271) compared to the control (*n* = 84,864) period (*p* = 0.0017). ED trauma team activation numbers remained unchanged. SVSAs (corrected for seasonality) also remained unchanged during control (*n* = 138) and pandemic (*n* = 129) periods, and no differences were observed for suicide methods, injury patterns, or length of hospital stay.

**Conclusion:**

Our data emphasize that a previously reported rise in psychological stress during the COVID-19 pandemic does not coincide with increased SVSA rates or changes in quality of SVSAs.

## Introduction

The COVID-19 pandemic imposed an unprecedented burden on health care systems worldwide. To effectively slow down the spread of SARS-CoV-2, governmental authorities decreed lockdown and shutdown measures of various degrees and lengths during 2020 and 2021 (1). While patients suffering from COVID-19 impacted intensive care units and hospital services, individuals and communities were additionally challenged by a rise in depression, anxiety, and acute stress symptoms resulting from quarantine and social distancing measures (2). Consistent data from the U.S. and Europe demonstrated an initial deterioration in mental health during the first months of the pandemic in March and April 2020, followed by a considerable degree of recovery during June, July, and August 2020 (3–5). Whether or not alternating states of mental health and depression may have influenced harmful behavior and suicide attempts, concerned authors since the beginning of the pandemic (6, 7), especially in the light of reports on increased suicide rates during previous healthcare crises like the SARS outbreak in Hong Kong in 2003 (8, 9).

As data on suicide rates during the pandemic slowly emerged, geographic differences became apparent. For example, while suicide rates in a majority of western countries, including Germany (10), did not increase but remain stable or decrease (11), other studies reported a rise in suicide attempts and completed suicides during the first months of the pandemic in Nepal, Italy and South Korea (12–14).

Japan also reported a rise in suicide numbers during the second wave of COVID-19 infections, following an initial decline (15). Data from the U.S. further showed ethnicity-based shifts in suicide rates during the pandemic in Maryland (16) and Connecticut (17).

While nonviolent suicide attempts (poisoning or overdosing) represent the majority of index suicide attempts, violent suicide attempts (e.g., hanging, cutting or piercing, jumping from great heights) represent the majority of completed suicides (18). Violent suicide attempts are often tied to injuries of the musculoskeletal system and have a higher prevalence among men (19). They were previously linked to ambient temperatures and a lack of sunshine hours (20, 21).

While most available studies focus on the number of suicides during the COVID-19 pandemic, reports on suicide quality remain scarce. In this study, we evaluated serious violent suicide attempts (SVSAs), defined as violent suicide attempts that would have been potentially lethal had it not been for rapid and effective emergency treatment (22). SVSAs were analyzed for suicide method, injury severity, and concomitant treatment strategies during 14 months amid the COVID-19 pandemic and a preceding 14-month control period in four Level-I Trauma Centers in Berlin, Germany.

## Materials and methods

### Study design and setting

#### Context/places

This retrospective study was conducted at four medical centers that fulfill the Level-I Trauma Center criteria, defined by the American College of Surgeons (ACS) and the German Trauma Society (DGU) (23). As part of a network of five Level-I Trauma Centers in Berlin, Germany, the four participating centers provide full 24/7 in house trauma care for the German capital and its adjacent urban areas.

#### Patient sample and key dates

**Figure 1:**
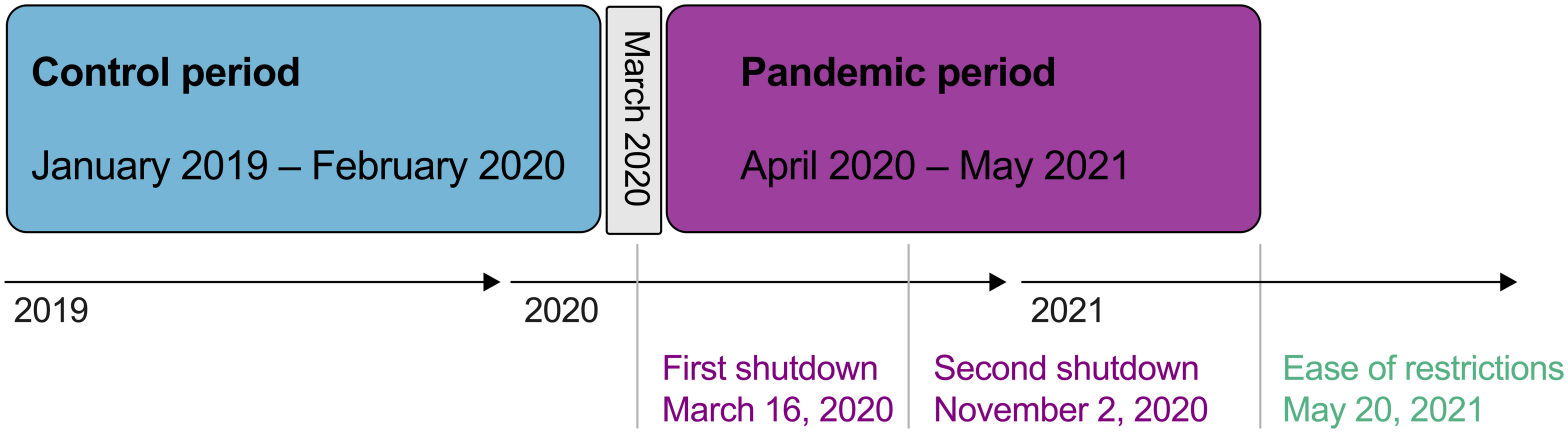
Longitudinal time chart of analyzed periods and COVID-19 related shutdowns implemented in Berlin, Germany during 2020 and 2021.

Absolute numbers for orthopaedic trauma patients, trauma team activations, and SVSAs admitted to the emergency departments (EDs) of the four participating Level-I Trauma Centers in Berlin, Germany, were retrospectively assessed. The following periods were defined as 14-month pandemic and control periods:

- January 1, 2019–February 29, 2020 (control).
- April 1, 2020–May 20, 2021 (pandemic; Figure 1).

On March 16, 2020, the German government and the federal states announced a first temporary shutdown of public life, including schools, universities, restaurants, and bars (24). After some restrictions were lifted during the summer of 2020, a second shutdown was imposed on November 2, 2020 (25). On May 20, 2021 shutdown measures were mostly lifted in Berlin, Germany, which marks the endpoint of the pandemic period in our analyses (Figure 1). March 2020 was excluded from the analyses to allow a clear discrimination between the control and pandemic period.

#### Sample frame and data collection

Investigators from each center retrieved data from hospital healthcare databases [retrospective chart review employing the convenience sampling approach (26)]. First, absolute numbers for orthopaedic trauma patients, trauma team activations, and SVSAs were evaluated. Second, all SVSA cases were reviewed in detail for parameters surrounding the quality of suicide attempts (Table 1). All patients were followed up until they were discharged from hospital or deceased.

**Table 1:** Characteristics of patients who committed serious violent suicide attempts (SVSAs) during a 14-month COVID-19 pandemic period and a 14-month control period in Berlin, Germany.

**Characteristic**	**Control**,	**Pandemic**,	***p*** **-Value** ^2^
	***N*** = **138**^1^	***N*** = **129**^1^	
Hospital I	52 (38%)	45 (35%)	0.2
Hospital II	28 (20%)	21 (16%)	
Hospital III	17 (12%)	28 (22%)	
Hospital IV	41 (30%)	35 (27%)	
Female	45 (33%)	51 (40%)	0.2
Male	93 (67%)	78 (60%)	0.2
Age	43.0 (32.0, 58.0)	40.0 (28.0, 56.0)	0.2
Alcohol consumption	19 (14%)	15 (12%)	0.6
Drug consumption	19 (14%)	27 (21%)	0.12
Regular substance abuse	32 (23%)	36 (28%)	0.4
Psychiatric diagnosis	93 (67%)	79 (61%)	0.3
Jump from great height	56 (41%)	55 (43%)	0.7
Height	10.0 (5.5, 13.0)	9.0 (6.0, 14.0)	0.7
Train collision	16 (12%)	10 (7.8%)	0.3
Traffic collision	4 (2.9%)	1 (0.8%)	0.4
Cutting/piercing	41 (30%)	44 (34%)	0.4
Strangulation	5 (3.6%)	9 (7.0%)	0.2
Other methods	8 (5.8%)	4 (3.1%)	0.3
>1 method	8 (5.8%)	6 (4.7%)	0.7
Face	17 (12%)	20 (16%)	0.5
Skull	15 (11%)	11 (8.5%)	0.5
Clavicle	8 (5.8%)	5 (3.9%)	0.5
Humerus	17 (12%)	19 (15%)	0.6
Olecranon	3 (2.2%)	6 (4.7%)	0.3
Radius/Ulna	17 (12%)	17 (13%)	0.8
Hand	8 (5.8%)	6 (4.7%)	0.7
Ribs	35 (25%)	35 (27%)	0.7
Sternum	9 (8.2%)	10 (9.3%)	0.8
O-C spine	7 (5.1%)	5 (3.9%)	0.6
C spine	10 (7.2%)	6 (4.7%)	0.4
T Spine	25 (18%)	22 (17%)	0.8
L Spine	34 (25%)	33 (26%)	0.9
Sacrum	20 (14%)	17 (13%)	0.8
Pelvis	35 (25%)	31 (24%)	0.8
Femur	18 (13%)	21 (16%)	0.5
Tibia/Fibula	18 (13%)	28 (22%)	0.061
Patella	1 (0.7%)	5 (3.9%)	0.11
Foot	30 (22%)	29 (22%)	0.9
Open fracture	36 (26%)	18 (14%)	0.014
Excessive hemorrhage	25 (18%)	16 (12%)	0.2
Amputation	6 (4.3%)	1 (0.8%)	0.12
Pharynx/Trachea	6 (4.3%)	4 (3.1%)	0.8
Neck arterial vessel	5 (3.6%)	4 (3.1%)	>0.9
Pneumothorax	38 (28%)	35 (27%)	>0.9
Hematothorax	15 (11%)	18 (14%)	0.4
Pulmonary contusion	30 (22%)	23 (18%)	0.4
Intrathoracal arterial vessel	3 (2.2%)	7 (5.4%)	0.2
Heart	4 (2.9%)	3 (2.3%)	>0.9
Liver	13 (9.4%)	17 (13%)	0.3
Spleen	14 (10%)	8 (6.2%)	0.2
Stomach/Bowel	9 (6.5%)	7 (5.4%)	0.7
Kidney	11 (8.0%)	7 (5.4%)	0.4
Peritoneum/omentum	9 (8.2%)	6 (5.6%)	0.4
Intraabd. art. vessel	7 (5.1%)	10 (7.8%)	0.4
Subdural haematoma	12 (8.7%)	9 (7.0%)	0.6
Subarachnoidal hemorrhage	13 (9.4%)	13 (10%)	0.9
Intracerebral hemorrhage/contusion	9 (6.5%)	5 (3.9%)	0.3
Injury Severity Score (ISS)	22.0 (8.0, 29.0)	17.0 (5.0, 34.0)	0.6
Surgical resucitation	2 (1.4%)	7 (5.4%)	0.094
Emergency surgery	86 (62%)	71 (55%)	0.2
Semi-elective surgery	12 (8.7%)	11 (8.5%)	>0.9
Elective surgery	7 (5.1%)	6 (4.7%)	0.9
Conservative treatment	22 (16%)	18 (14%)	0.6
ED treatment	9 (6.5%)	16 (12%)	0.1
Death in ED	6 (4.3%)	3 (2.3%)	0.5
Death in ICU	9 (6.5%)	12 (9.3%)	0.4
Death in operating theater	1 (0.7%)	4 (3.1%)	0.2
Discharged home	22 (16%)	26 (20%)	0.4
Transferred to other facility	100 (72%)	84 (65%)	0.2
Days spent in hospital	11.0 (3.0, 24.2)	6.5 (2.0, 21.0)	0.12
Days spent in ICU	5.0 (2.0, 12.0)	3.0 (2.0, 9.0)	0.049

*C spine, cervical spine; ED, emergency department; ICU, intensive care unit; L spine, lumbar spine; ISS, Injury Severity Score; O-C spine, occipito-cervical spine; T spine, thoracic spine*.

*^1^n (%); Median (IQR)*.

*^2^Pearson's Chi-squared test; Fisher's exact test; Wilcoxon rank sum test*.

#### Inclusion criteria

All orthopaedic trauma patients (defined as patients who were primarily treated by an orthopaedic trauma surgeon in the ED), and all cases where the trauma team was activated [initiated if patients met the Grade of Recommendation B-criteria (GoR-B) defined by DGU^®^ (e.g. fall/jump from great heights, penetrating or gunshot injuries, high-velocity traffic accidents) (27)] were included. All SVSAs were included if the provided information in the medical chart indicated a suicide attempt or where a suicide intention leading to the injury could not be ruled out (e.g. in case of schizophrenia or bipolar disorder).

#### Exclusion criteria

For orthopaedic trauma patients and trauma team activations no exclusion criteria were required. For SVSAs we excluded patients who denied suicidal intentions and where an unintentional accident was plausible after ruling out mental health conditions.

#### Ethics

Ethical approval (EA1/082/20) was obtained from the local ethics committee, Ethikkommission Charité – Universitätsmedizin Berlin.

### Statistical analysis

Data were analyzed primarily descriptively. Incidences were presented as absolute counts within the period of observation. For both the pandemic and the control period, specific characteristics of SVSAs were calculated and compared in an exploratory manner. In case of continuous and ordinal data, mean, median, interquartile range and standard deviation (SD) were calculated as well as the *p*-value from a Wilcoxon signed-rank test. For binary data, corresponding proportions were calculated as well as the *p*-value from Pearson's Chi-Squared test, or Fisher's exact test if the expected count in any cell was below 5. To compare variables during the pandemic vs. control period, odds ratios (ORs) with 95% confidence intervals (CIs) were calculated. To allow for scale-independent comparisons between ORs of continuous variables, variables were standardized to a mean of 0 and an SD of 1.

A central aim of the analysis was the comparison of monthly incidences of SVSAs between the control and the pandemic period. To control for seasonal trends, a regression approach was chosen. The limited time period and the relatively small incidence raised concerns regarding the appropriate model fit of simple linear regression. Therefore, a sensitivity analysis also considering regression models for count data, i.e., poisson and negative binomial regression were fitted, too, and the results of each regression approach were compared.

All observations during the defined time frame within the participating Level-I Trauma Centers were included in the analysis, so no sample size calculation was conducted. Instead, an exemplarily power analysis was used to justify the chosen time frame: based on the assumption of a monthly incidence of ~10 SVSAs, the 95% CI of the pairwise difference between pre- and post-pandemic months would have an expected width of 1.0 SVSA per month.

## Results

### Absolute numbers and seasonality

**Figure 2:**
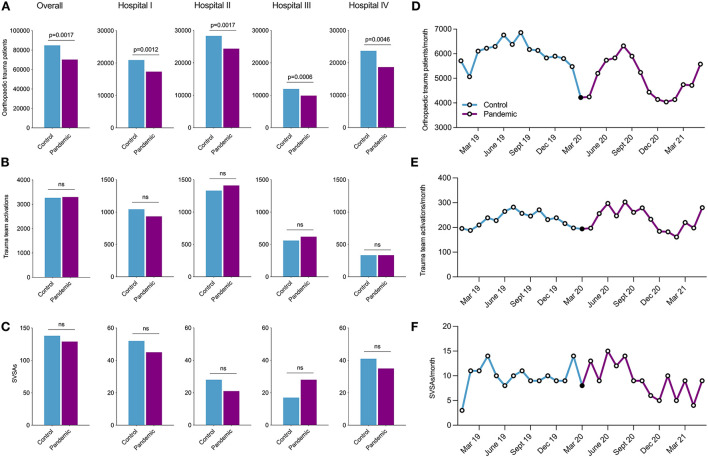
Overall and hospital-specific distribution of **(A)** orthopaedic trauma patients, **(B)** trauma team activations, and **(C)** SVSAs during the 14-month control (blue) and pandemic period (purple). Longitudinal distribution of **(D)** orthopaedic trauma patients, **(E)** trauma team activations, and **(F)** SVSAs per month during the control (blue) and pandemic period (purple). March 2020 (black dot) was excluded from the analyses. SVSAs, serious violent suicide attempts.

During the pandemic period, significantly fewer orthopaedic trauma patients presented to the ED during the pandemic (*n* = 70,271) compared to the control (*n* = 84,864) period (*p* = 0.0017). Subgroup analyses confirmed this pattern for each center (hospital I, *p* = 0.0012; hospital II, *p* = 0.0017; hospital III, *p* = 0.0006; hospital IV, *p* = 0.0046; Figure 2A). Furthermore, the longitudinal development of orthopaedic trauma patients per month unveiled a decrease in cases, especially during spring and autumn 2020, when lockdown measures were in effect in Berlin, Germany (Figure 2B).

Trauma team activation numbers remained unchanged during control and pandemic periods, with 3.267 and 3.298 cases, respectively (Figure 2C). Although overall numbers of trauma team activation did not differ, decreases were observed during shutdown periods (Figure 2D). Lastly, SVSAs also remained unchanged when comparing control (*n* = 138) and pandemic (*n* = 129) periods (Figure 2E). Monthly SVSAs were evenly distributed between February 2019 and August 2020, followed by a slight decline between September 2020 and May 2021 (Figure 2F). Interestingly, SVSAs decreased in all hospitals during the pandemic period, apart from hospital III (CBF), where an increase, yet not significant, was observed (*p* = 0.1270; Figure 2E).

Controlling for seasons, linear regression (*p* = 0.427), poisson regression (*p* = 0.446), and negative binomial regression (*p* = 0.446) models showed no differences between control and pandemic periods.

Additionally, we compared two calendar-matched 12-month periods from April 2019-March 2020 (control II) to April 2020-March 2021 (pandemic II) and found similar results, where linear regression (*p* = 0.523), poisson regression (*p* = 0.595), and negative binomial regression (*p* = 0.595) models showed no differences between control II and pandemic II periods.

### Quality of SVSAs

#### Demographics and suicide motivation

After assessing absolute numbers of SVSAs, changes in quality and severity of SVSAs during the control and pandemic period were evaluated. No relevant differences were observed between the two periods for all variables assessed (Table 1).

Women accounted for 33% (*n* = 45) of SVSAs during the control and for 40% (*n* = 51) of SVSAs during the pandemic period. The median (IQR) age was 43 (29;30) years during the control and 40 (28;31) years during the pandemic period. Alcohol and drug intoxications in association with SVSAs were observed during control (both 14%, *n* = 19) and pandemic (alcohol 12%, *n* = 15; other drugs 21%, *n* = 27) periods. Regular substance abuse was seen in 23% (*n* = 32) of cases during the control and in 28% (*n* = 36) of cases during the pandemic period.

**Figure 3:**
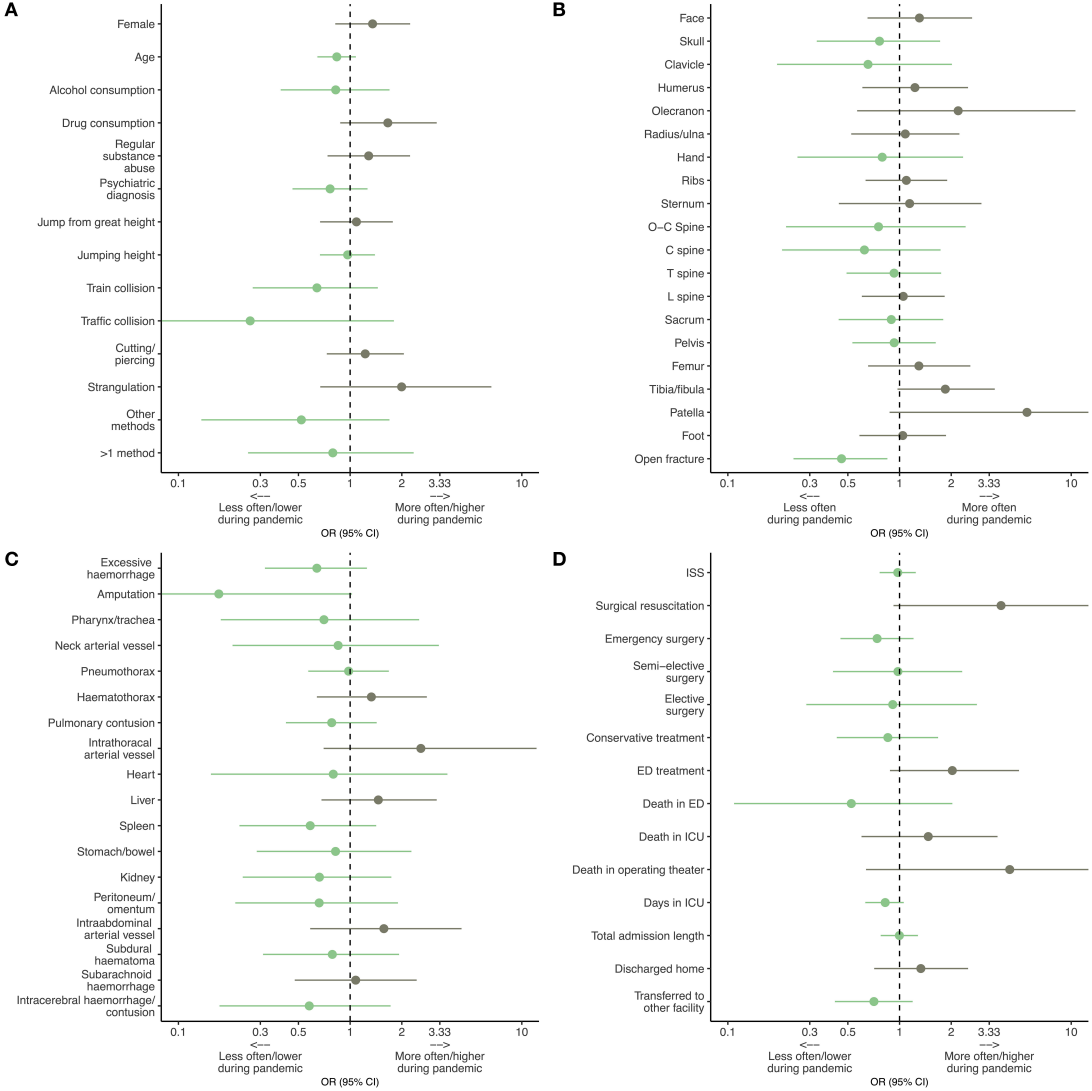
Odds ratios (ORs) for **(A)** demographics and suicide methods, **(B)** osseous injury patterns, **(C)** non-osseous injury patterns, **(D)** Injury Severity Score (ISS), treatment and outcomes of patients who undertook serious violent suicide attempts (SVSAs) during the COVID-19 pandemic and a proceeding control period. Green confidence intervals (95% CIs) indicate an event happening less often during the pandemic, whereas gray 95% CIs represent events happening more often during the pandemic. To obtain symmetric CIs, a log-scaled x-axis was used (few, very wide CIs were cut for proper display). ED, emergency department; C spine, cervical spine; ICU, intensive care unit, L spine, lumbar spine, O-C spine, occipito-cervical spine, T spine, thoracic spine.

During the control period, psychiatric diagnoses were evaluated for 67% (*n* = 93) and during the pandemic for 61% (*n* = 79) of patients committing SVSAs (Figure 3A and Table 1).

#### Methods of SVSAs

With 41% (*n* = 56) and a median (IQR) height of 10 (5.5;13) meters and 43% (*n* = 55) and a median height of 9 (6;14) meters, jumps from great heights were the most common suicide method observed during control and pandemic periods, respectively. Cutting and piercing injuries were seen in 30% (*n* = 41) and 34% (*n* = 33) of cases during the control and pandemic period. SVSAs including subway train collisions were observed in 12% (*n* = 16) of cases during the control and in 7.8% (*n* = 10) of cases during the pandemic period. Strangulations were evident in 3.6% (*n* = 5) of cases during the control and in 7.0% (*n* = 9) of cases during the pandemic period. SVSAs including more than 1 method were observed in 5.8% (*n* = 8) of cases during the control and in 4.7% (*n* = 6) of cases during the pandemic period (Figure 3A and Table 1).

#### Injury patterns following SVSAs

Please refer to Table 1 and Figures 3B, C for a detailed overview of osseus and non-osseus injury patterns. No relevant changes were observed apart from open fractures, which were seen significantly less often during the pandemic period with 14% (*n* = 18) compared to the control period with 25% (*n* = 36) (*p* = 0.014). In addition, the median (IQR) ISS during the control period was 22 (8, 31) compared to 17 (5, 32) during the pandemic period.

#### Treatment of SVSAs

Resuscitation surgery was performed twice during the control period and seven times during the pandemic period. Sixty-two percent (*n* = 68) and 55% (*n* = 71) of patients required emergency surgery and 8.7% (*n* = 12) and 8.5% (*n* = 11) semi-elective and 5.1% (*n* = 7) and 4.7 (*n* = 6) elective surgery during the control and pandemic period. Sixteen percent (*n* = 22) and 14% (*n* = 18) of patients were treated conservatively and 6.5% (*n* = 9) and 12% (*n* = 16) of patients received ED treatment during respective control and pandemic periods (Figure 3D and Table 1).

#### Outcomes following SVSAs

Six patients died in the ED, nine in the ICU and one in the operating theater (11.6%; *n* = 16) during the control period and three in the ED, 12 in the ICU and four in the operating theater during the pandemic period (14.7%; *n* = 19).

Sixteen percent (*n* = 22) of patients could be discharged to their homes during the control and 20% (*n* = 26) during the pandemic period. The remaining patients (72%; *n* = 100 during the control and 65%; *n* = 84 during the pandemic) were transferred to other specialized psychiatric or rehabilitation facilities.

Overall, SVSA patients spent a median (IQR) of 11 (3;24) days in hospital during the control period and 6.5 (2;21) days during the pandemic period. This included a median (IQR) of five (2;12) and three (2;9) days in the ICU during respective periods (Figure 3D and Table 1).

## Discussion

The present study demonstrates a constant rate of SVSAs and trauma team activations during a 14-month period during the COVID-19 pandemic compared to a preceding control period. Furthermore, we were able to show that the quality of SVSAs, including suicide method, injury severity, type of treatment, and admission time did not change between the two assessed periods. Yet, the total number of orthopaedic trauma patients decreased significantly, in the EDs of four Level-I Trauma Centers in Berlin, Germany.

While previous studies reported an increase in depression and suicidal ideation during the COVID-19 pandemic (28, 31, 33, 34), most high volume data on actual suicide attempts failed to show a concomitant increase in suicides (10, 11, 32, 35–37). Our data are in line with these findings and we were able to show that violent suicide attempts did not increase during the first 14 months of the pandemic in Berlin, Germany. Nevertheless, few studies with limited patient numbers have demonstrated an increase in suicide rates during specific periods amid the COVID-19 pandemic in 2020 (12–15). Previous health crises, including the SARS epidemic in Hong Kong and the Ebola virus epidemic in Guinea led to an increase in suicide attempts and completed suicides (39), however, thus far this has not been the case for the COVID-19 pandemic.

While previous health crises, including the SARS epidemic in Hong Kong, and the Ebola virus epidemic in Guinea led to an increase in suicide attempts and completed suicides (38), thus far this has not been the case for the COVID-19 pandemic.

Reasons for this may have been prompt economical support programs, implemented in Germany, which helped to reduce financial losses for individuals and companies (39). While recessions have previously been linked to increased suicide rates, the effect seemed to be less pronounced in countries with larger social welfare spending (40–42).

Second, access to healthcare during the COVID-19 pandemic was perceived as reasonably good among the German population (43). Notably, access to healthcare has previously also been shown to be a crucial factor in suicide prevention (44, 45).

It is however too early to finalize conclusions on suicide rates during the COVID-19 pandemic, which is still ongoing in various countries worldwide. Although the majority of currently published studies on the topic could not show an increase in suicide attempts and completed suicides during the pandemic (10, 11, 32, 35–37), suicides may still rise with a certain delay, as previously seen in periods following natural catastrophes in the US (46).

In a Swedish registry-based study, cutting or piercing accounted for 35.6% of violent index suicide attempts and only for 4.8% of completed violent suicides (18). In line with these findings, we observed cutting or piercing in 29.7% of cases (*n* = 41) during the control, and in 34.1% of cases (*n* = 44) during the pandemic period.

While we saw jumps from great heights in 40.6% (*n* = 56) of the study population during the control and in 42.6% (*n* = 55) of cases during the pandemic period, they only accounted for 9.5% of violent index attempts and 8% of completed violent suicides in the Swedish registry-based study (18). This marked difference may be attributed to our study population, exclusively derived from an urban environment. It was previously described that the accessibility to lethal methods, including living in higher buildings, determines the method of suicide (47).

One study from South Korea reported a decline in violent suicide attempts (“self-harm injury”) from 82.6% before the pandemic (January–October 2019) to 30% during the pandemic (January–October 2020), while they observed a rise in drug overdoses from 13.3 to 66.6% (14).

Marked differences can be observed between suicide completers, where hanging is the most prevalent method, and suicide attempters, where poisoning is most commonly observed (48). The method chosen at the index suicide attempt also predicts the chances of suicide completion at a later point. Suicide completers most commonly chose the same method during an index suicide attempt (90% for hanging) (49).

Effects of seasonality, weather, and temperature on suicide rates have been extensively reported in previous decades. While suicide peaks were described for spring and late summer in the 1960s−1980s (50, 51) more recent data indicate that seasonal patterns have diminished regarding suicide rates. Reasons identified are an increased intensity of communal life and technological developments (52–54). Accordingly, our data showed no differences in SVSA rates, when controlled for seasonality.

While incidences of neuro-psychiatric diagnoses rose during the COVID-19 pandemic, in both, patients suffering from COVID-19 (55) and healthy individuals (28), early reports also suspected a direct translation into suicide attempts (30, 56). In a commentary, Sher reported a high probability of patients suffering from post-COVID-19 syndrome to show an increase in suicidal ideation and behavior due to long-term psychiatric, neurological, and physical illness (57). To date, most data could not confirm concerns about increased suicide rates during the COVID-19 pandemic, raised in various editorials, reviews and commentaries.

Reporting on suicidal behavior must be conducted with caution, as misleading data and sensational media coverage may considerably influence suicide intentions and imitations. Scientific reporting is obligated to be conducted in a precise and balanced manner and should focus on facts rather than speculations (29, 58).

This study has several limitations. We were able to analyze ED records of four Level-I Trauma Centers, yet, our data is merely representative for the urban area of Berlin, Germany. While conclusions could be relevant for other capital regions, they may fall short when looking at provincial areas or countries with alternate urban environments (47). Second, shutdown measures varied greatly between regions and countries. Therefore, data may be interpreted with caution and should always be put in perspective to available international evidence. Third, the retrospective character of our study does not allow assumptions about causation, but rather about associations between the COVID-19 pandemic and suicidal behavior. Finally, this study only covered the first 14 months of the COVID-19 pandemic and conclusions for the pandemic as a whole are not valid at this point. Most studies reporting on suicide numbers and rates have however covered shorter time periods and long-term data is still scarce.

To address a potential selection bias based on hospital location, we included four Level-I Trauma Centers that geographically form a string spanning from West to East Berlin. We thereby anticipated to minimize a geographical or socioeconomical selection of patients.

Retrospective chart review studies often include a relevant documentation bias (data entry, data collection, and data quality assurance). We employed a standardized data retrieval sheet (Microsoft Excel) for all four participating centers to ensure obtained data are of similar quality. Following the guide of Gearing et al. (26), sections for each variable in the data retrieval sheet were created using simple and unambiguous response options. Nonetheless, documentation of e.g., alcohol consumption or history of mental illness is highly dependent on the documenting physician and may be a relevant source of bias.

The COVID-19 pandemic may have just surpassed its peak and new virus variants, including Omicron, seem to cause milder symptoms in affected individuals compared to previous virus strains. Nonetheless, lockdown and shutdown measures are still in place in numerous countries to date. Recent data indicated that restrictions and subsequent self-isolation related to COVID-19 correlated with a rise in psychiatric diagnoses. Whether this would also impact suicide attempts was discussed by a number of authors.

This study was able to demonstrate that violent suicide attempts did not change in quantity nor in quality during the COVID-19 pandemic compared to a control period prior to the SARS-CoV-2 outbreak in 2019/2020. These data may help to map out “collateral damage” scenarios resulting from social distancing, while preparing for future pandemics. Whether current lockdown and shutdown measures have an impact on long-term mental health and suicidal behavior remains to be seen.

## Data availability statement

The raw data supporting the conclusions of this article will be made available by the authors, without unduereservation.

## Ethics statement

The studies involving human participants were reviewed and approved by Ethikkommission der Charité – Universitätsmedizin Berlin. Written informed consent from the participants' legal guardian/next of kin was not required to participate in this study in accordance with the national legislation and the institutional requirements.

## Author contributions

TM and SM designed and conceived the study. TM, TT, ADD, ED, GR, MZ, RC, MK, PW, and FF provided, selected, assembled, analyzed, and interpreted data. DZ and LSH provided statistical, graphical and software support. TT, US, TF, DG, NS, AR, and SM curated data and provided project administration. TM drafted the original manuscript. All authors agree to be accountable for the work as a whole and ensure that questions related to the accuracy or integrity of any part of the work are appropriately investigated and resolved. All authors critically reviewed and edited the final manuscript and have read and confirmed the final version, submitted to Frontiers in Psychiatry.

## Conflict of interest

The authors declare that the research was conducted in the absence of any commercial or financial relationships that could be construed as a potential conflict of interest.

## Publisher's note

All claims expressed in this article are solely those of the authors and do not necessarily represent those of their affiliated organizations, or those of the publisher, the editors and the reviewers. Any product that may be evaluated in this article, or claim that may be made by its manufacturer, is not guaranteed or endorsed by the publisher.
